# Gestational age and sex interaction and risk for autism spectrum disorder in extremely preterm newborns: an 18‑month follow‑up study

**DOI:** 10.1192/j.eurpsy.2024.588

**Published:** 2024-08-27

**Authors:** L. Pina-Camacho, J. Merchan-Naranjo, E. Rodriguez-Toscano, L. Martin, C. Romero, L. Boada, S. Zeballos, M. Arriaga, D. Blanco-Bravo

**Affiliations:** ^1^Department of Child and Adolescent Psychiatry, Institute of Psychiatry and Mental Health, Hospital General Universitario Gregorio Marañon, CIBERSAM, IiSGM, School of Medicine, Universidad Complutense; ^2^Neonatology Service, Hospital General Universitario Gregorio Marañon, IiSGM, School of Medicine, Universidad Complutense, Madrid, Spain

## Abstract

**Introduction:**

Extremely preterm newborns - EPTN (born ≤28 weeks gestational age) are at increased risk of developing autism spectrum disorders (ASD). Demographic and perinatal risk factors associated with ASD risk in EPTN are understudied.

**Objectives:**

(i) In EPTN and born at full-term healthy controls (HC), to characterize the emergence of ASD traits and autistic symptom load at age 18 months; (ii) in EPTN, to identify the influence of perinatal characteristics such as sex and gestational age on autistic symptom load at corrected-age 18 months.

**Methods:**

Observational, longitudinal, prospective, 18-month follow-up study. We recruited a cohort of n=113 EPTN and n=47 HC (the PremTEA cohort); n=57 EPTN and n=42 HC successfully completed the 18-month follow-up visit. We assessed autistic symptom load & risk at 18 months using the M-CHAT-R/F questionnaire. For all EPTN and HC, we collected demographic and perinatal data. Using GLMs, we assessed, in EPTN, the association between demographic/perinatal variables and 18-month autistic symptom levels.

**Results:**

At 18 months, EPTN children showed higher autistic symptom levels than HC (M-CHAT-R/F score, mean (SD) [range] = 2.21 (3.23) [0-12] in EPTN vs. 0.33 (0.57) [0-2] in HC; d=.873, p=.001. In EPTN, we identified differences by gestational age and sex in autistic symptom levels at 18 months (aR^2^=0.517, p=.006). In particular, female EPTNs born with lower gestational age showed higher autistic symptom load at age 18 months.

**Conclusions:**

Our findings support the need for early screening of ASD symptomatology in EPTN infants, particularly in higher-risk subgroups, such as female patients born with lower gestational ages.

**Disclosure of Interest:**

None Declared

